# The effectiveness and acceptability of face-to-face rehabilitation for patients with Long Covid who were not hospitalised with their acute infection: a mixed-methods study comprising a randomised controlled trial (RCT) with embedded qualitative component

**DOI:** 10.1186/s13063-025-09419-z

**Published:** 2026-01-21

**Authors:** Kate Kontou, Enya Daynes, Sally J. Singh, Rachael A. Evans, Nikki Gardiner, Emma Chaplin, Matthew Richardson, Nicolette Bishop, Jennifer Creese, Nicola Bateman, Adam Wright, Elga Zivtins, Linzy Houchen-Wolloff

**Affiliations:** 1 CERS, Respiratory BRC, Glenfield Hospital, University Hospitals of Leicester NHS Trust, Leicester, UK; 2 Respiratory Sciences, Glenfield Hospital, University of Leicester, Leicester, UK; 3 Glenfield Hospital, University Hospitals of Leicester NHS Trust, Leicester, UK; 4 Loughborough University, Epinal Way, Loughborough, UK; 5 Department of Population Health Sciences, George Davis Centre, University of Leicester, 15 Lancaster Road, Leicester, UK; 6 School of Business, University of Leicester, Brookfield, 266 London Road, Leicester, UK; 7 Patient Advisory Group, Leicester, UK

**Keywords:** Long Covid, Rehabilitation, Randomised controlled trial, Mixed methods

## Abstract

**Background:**

Long Covid is a term used to describe a multisystem condition that presents with a myriad of physical and psychological symptoms that continue or develop after acute COVID-19. Long Covid is a significant public health problem because of the nature of the illness, its negative impfact on everyday functioning, and the healthcare inequalities evident in access and experience, notably in terms of ethnicity and socioeconomic status. Evidence in patients hospitalised with their acute infection suggests exercise-based rehabilitation could be helpful to improve exercise tolerance, respiratory symptoms, fatigue, and cognition; however, research is needed to determine whether exercise-based rehabilitation is effective and acceptable for patients with Long Covid who were not hospitalised.

**Methods:**

This mixed-methods study comprises a single-centre, randomised controlled trial to determine whether face-to-face rehabilitation increases exercise capacity compared to usual care alone in non-hospitalised patients with Long Covid, with embedded qualitative components to explore intervention acceptability in the context of healthcare inequalities. Usual care is as defined by the National Institute for Clinical Excellence (NICE) Covid-19 guidance.

The rehabilitation intervention will take place twice a week for 6 weeks and will combine symptom-titrated exercise with self-management education. The proposed sample size of 56 for the randomised controlled trial is calculated on the primary outcome of Incremental Shuttle Walking Test (ISWT) distance, with a change of 50metres (m) at 90% power, a standard deviation of 17 m, and a 0.05 type 1 error.

**Discussion:**

A mixed-methods approach has been chosen as quantitative data alone would be insufficient to answer the research question, and mixing the data will enable a more comprehensive understanding and ensure there is an equal focus on outcomes and experiences of a face-to-face exercise-based rehabilitation programme. A healthcare inequalities lens will explore who may be under-represented, with the qualitative work providing further evidence as to why this may be the case. It is recognised that meeting recruitment targets in the context of reducing referral rates and funding for Long Covid services may prove challenging.

**Trial registration:**

ISRCTN trial registry (ISRCTN33340595). Registered on 30 September 2024.

**Supplementary Information:**

The online version contains supplementary material available at 10.1186/s13063-025-09419-z.

## Introduction

### Background and rationale {6a}

Long Covid, a term preferred by patient advocates, is defined by NICE as follows: ‘a multi-system condition with a range of debilitating symptoms — signs and symptoms continue or develop after acute COVID-19, continue for more than 4 weeks, and are not explained by an alternative diagnosis’ [1].

Patients with Long Covid present with a myriad of symptoms that can include fatigue, shortness of breath, headache, cognitive dysfunction (‘brain fog’), chest pain, muscle and joint pains, cough, disturbed sleep, and psychological symptoms, such as anxiety and depression [2, 3]. These symptoms are often unpredictable and concurrent, which can significantly negatively impact everyday functioning and occupation [4]. It is difficult to substantiate the number of cases of Long Covid in the UK, especially in the absence of a diagnostic test; however, the most recent national estimates suggest that around 2 million people in England and Scotland were experiencing symptoms as of March 2024 [5].

The nature of the illness, together with its prevalence, suggests that Long Covid is a significant public health problem [5]. There is a limited evidence base to inform which interventions may be effective; however, evidence on exercise-based rehabilitation has demonstrated positive effects in improving exercise tolerance, respiratory symptoms, fatigue, and cognition [6–9]. Most rehabilitation studies to date have been observational cohorts, so the effects of natural recovery versus the benefits of rehabilitation are unknown. Furthermore, previous studies have focused on hospitalised cohorts as opposed to patients who had their acute Covid managed out of hospital (‘community managed’) [10, 11].

Provision of care can vary widely between and within countries. In the UK, for example, there is guidance from NICE, the NHS Plan for Improving Long Covid services, and Long Covid-specific commissioning guidelines [12–14]. Interpretation of these guidelines varies regionally and nationally [15]. Hospitalisation status is increasingly being recognised as an inequality in itself, with access to Long Covid follow-up services being worse among patients who were never admitted to hospital [14], in comparison to hospitalised patients who have typically received more proactive follow-up [15, 16]. There is qualitative evidence to suggest that those not hospitalised may feel unsupported and disbelieved by health services [17] and some remain unsure of how and where to seek healthcare support [18].

There is ongoing research into inequalities in Long Covid care, specifically with regard to ethnicity and socioeconomic groups [19]. Early in the pandemic, it was recognised that some ethnic groups were disproportionately affected by Covid-19 [20], and, more recently, research has shown Long Covid is strongly associated with socioeconomic deprivation. This was highlighted in a recent study, which found that the odds of developing Long COVID are 46% higher on average in the most deprived areas compared to the least [21].

The proposed mixed-methods research design includes an exploratory randomised controlled trial with an embedded qualitative evaluation with framed questions to investigate the acceptability of this intervention and healthcare inequalities in access and experience. This study will provide guidance for service structure and design; building on the considerations outlined for this specific patient population [22]. It is anticipated that the findings will be transferrable and help inform the development and delivery of effective rehabilitation for other long-term health conditions.

### Objectives {7}

#### Primary objective

The primary objective of this study is to determine whether face-to-face rehabilitation increases maximal exercise capacity compared to usual care alone in nonhospitalised patients with Long Covid

#### Secondary objectives

• Determine the acceptability of the intervention based on participant’s engagement.
• Seek to understand patients’ perceptions of the barriers and facilitators to face-to-face exercise-based rehabilitation in people with Long Covid.
• Explore the effectiveness of a face-to-face rehabilitation programme on other symptoms commonly associated with Long Covid to include fatigue, breathlessness, reduced physical activity, health-related quality of life (HRQoL), anxiety, depression, and pain.
• Use a healthcare inequalities lens to explore which demographic groups may be under-represented within Long Covid rehabilitation, with the qualitative work providing further evidence as to why this may be the case.

#### Exploratory objectives

• Optional blood tests will investigate the effect of face-to-face rehabilitation vs. usual care on systemic and cellular markers of immune ageing and immune-mediated inflammation including (but not limited to) T-cell surface and intracellular phenotype analysis (with or without stimulation) and circulating biomarkers such as antibodies, cytokines, or other circulating proteins.
• Optional heart rate monitoring will investigate the effect of face-to-face rehabilitation vs. usual care on heart rate variability.

## Methods

### Trial design and setting {8 and 9}

**Fig. 1:**
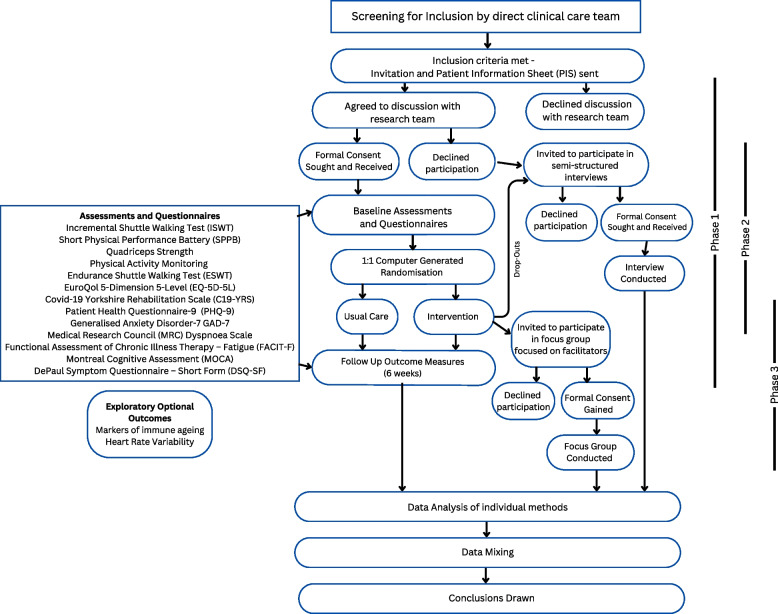
Study flow chart {13}

This is a single-centre mixed-methods study comprising an exploratory randomised controlled trial (RCT) with an embedded qualitative component to explore the effectiveness and acceptability of face-to-face rehabilitation for patients with Long Covid who were not hospitalised with their acute infection. Figure 1 provides an overview of the study processes.

This study will be conducted at the University Hospitals of Leicester (UHL) NHS Trust. Recruitment will take place at the outpatient Long Covid Clinic at UHL. Posters about this trial will be displayed within clinical waiting areas (e.g. Long Covid clinic). Recruiting directly from this medically led clinic at the point of rehabilitation referral will ensure that all participants have had a comprehensive multidisciplinary team (MDT) review, are medically optimised, and have been deemed medically appropriate to complete an exercise-based rehab programme. Ongoing medical support will be available for patients for the duration of the study.

### Participants – eligibility criteria {10}

Individuals must be willing to attend the face-to-face rehabilitation programme to participate in the RCT element of this study. Participants who are not willing or able to attend will be offered the opportunity to partake in the qualitative semi-structured interviews (phase 2) of this study.

Participants are eligible if they are over the age of 18 years old, were NOT admitted to hospital during the acute phase of their Covid infection, have a clinician diagnosis of Long Covid (from a dedicated Long Covid assessment clinic), have ongoing symptoms that may be modifiable by a rehabilitation programme (such as breathlessness, fatigue, or reduced exercise tolerance), and are willing and able to provide informed consent.

Individuals will be excluded from the study if exercise is contraindicated as outlined in the American College of Sports Medicine guidance [23], further investigation/management for an unstable comorbidity is required, exercise-based rehabilitation has been attended/completed in the preceding 6 months, hospital admission was required during their most recent acute SARS-CoV-2 infection, they have severe debilitating fatigue (home bound or bed bound) that worsens with activity — regardless of formal post-exertional malaise/myalgic encephalomyelitis (ME) diagnosis, or they are not willing or unable to provide consent.

### Who will take informed consent? {26a}

Consent will be sought and received by a member of the study team who has undergone appropriate consent training and is delegated to do this. Seeking informed consent will include explanations of the purpose of the research, the procedures of the study, any risks/benefits of participating in the research, and the participants’ right to withdraw at any point of the research. The participant will be able to ask any questions they have, and a participant information sheet written in lay language will be used to support this discussion.

### Additional consent provisions for collection and use of participant data and biological specimens {26b}

The RCT consent form will include optional tick boxes to consent for the additional exploratory outcome measures of blood inflammatory markers and heart rate variability and a ‘consent to be contacted for focus groups/interviews’ tick box. There will be a separate consent form to consent patients to the qualitative elements of the study.

Should patients choose to conduct their qualitative interview or focus group via remote means (telephone or videocall), informed consent may take place remotely, and an adapted ‘Remote Qualitative Consent form’ will be used. This will include a statement of fact (i.e. ‘I confirm that the participant has read and understood the PIS……’, ‘I confirm that the participant has had the opportunity to ask questions…’). The statements confirm that each section has been discussed, understood, and agreed with by the participant. This is then signed and dated, with a copy sent to the participant for their records by email/post.

Participants who consent to be contacted, but then decline to consent to the RCT element, will be asked whether they would be willing to participate in a semi-structured interview.

### Interventions {11d}

#### Phase 1 - The RCT

Both groups will receive the usual standard of care as defined by the National Institute for Clinical Excellence (NICE) COVID-19 guidance [24]. Implementing a twice-a-week rehabilitation programme for 6 weeks or usual care will not require alteration to usual care pathways (including the use of any medication), and these will continue for both trial arms. Participants will not be able to take part if they are currently taking part in another intervention research study.

### Face-to-face rehabilitation (intervention) {11a and 11c}

Individuals will complete an individualised symptom-titrated programme of exercise, education, and self-management for 6 weeks. The programme will comprise two supervised sessions per week delivered by healthcare professionals.

Six weeks have been purposefully selected as the intervention period to align with current clinical provision in Leicester. Aligning the intervention period with that used clinically will consolidate clinical expertise and support implementation.

The rehabilitation programme will consist of aerobic exercise (i.e. treadmill/ground walking at 80% of the incremental shuttle walk test (ISWT) speed where tolerated and lower-limb cycling on a static bike) and resistance exercise training (upper and lower limb strength exercises). The exercise intensity and duration will be tailored to the individual’s current abilities, assessed in their first visit.

In addition to the supervised sessions, patients will be asked to perform home-based exercise sessions which align with the supervised sessions to include the following: daily walking, three aerobic exercise sessions, and one resistance exercise session each week. Participants will be asked to record their progress in a self-reported diary, which will be used in conjunction with self-reported symptoms at the face-to-face sessions for the delivering clinicians to monitor symptoms and guide exercise modifications and progression.

Each rehabilitation session will include an educational discussion (approx. 30–60 min) delivered by a member of the multidisciplinary team. These discussions will be facilitated by information sheets saved from the Your COVID Recovery (YCR) website repository. Topics covered will include the following: getting moving again, managing activities of daily living, breathlessness, fatigue management, and recognising symptoms of worsening in response to exercise, fear and anxiety, mood and coping, memory and concentration, cough, eating well, sleep hygiene, goal setting, headaches, managing symptom exacerbation and fluctuations, and returning to work. At the end of each session, there will be an opportunity for discussion and question and answer.

Signs of post-exertional malaise and post-exertional symptom exacerbation will be monitored using the DePaul Symptom Questionnaire–Short Form (DSQ-SF) [25], modified Borg breathless scale [26], and Borg rate of perceived exertion (RPE) [27], and results were discussed by the healthcare professional with the participant at the start of each session. Adjustments to the exercise programme, or onward referral, will be made as necessary, and patients may be removed from the study if adverse events occur.

Clinical staff responsible for delivering the intervention have become experts in this now well-established intervention. Training will be provided to any new staff, and a competency scheme is in place to ensure there is standardisation of care.

### Usual care (control) {6b}

Participants in the control arm will receive usual care for 6 weeks. Routine clinical care will continue, such as medical follow-up, mental health service provision, and other specialist services.

#### Phase 2 – one-to-one semi-structured interviews

Interviews will be conducted by the lead researcher with those that declined (elected not to participate in the intervention at all) and those that dropped out of the programme (commenced but did not complete the intervention) to explore why people did not attend/complete in order to develop an understanding of the barriers to attendance. The data gathered from these interviews will help to identify and explore any healthcare inequalities that may be evident and whether there are any differences in the demographics of those who choose not to attend/complete. These will take place either face to face or virtually (by telephone or video conference) depending on the participants’ preference. It is expected that the interviews will take between 45 and 60 min depending on the responses.

The acceptability framework will inform the topic guide [28]; however, the nature of semi-structured interviews offers the researcher agency to adapt the interview for each participant depending on the answers provided.

#### Phase 3 - face-to-face focus groups

Focus groups will be conducted by the lead researcher (K. K.) who will act as the primary moderator and a research associate who will act as an assistant moderator to take field notes. Completers of the intervention group will be invited to take part in a focus group of around 8–10 participants. The aim of the focus groups will be to explore the facilitators to attendance and any healthcare inequalities that may be apparent. The acceptability framework will inform the topic guide, which will be evaluated by the patient advisory group prior to finalising. It is expected the focus group will take between 60 and 90 min.

Community language interpreters will be provided as required in any of the study phases.

All interviews and focus groups will be audio recorded with permission and transcribed verbatim removing all identifiable information to ensure anonymity.

Reimbursement of expenses will be made to those who travel to take part in either the focus group or a semi-structured interview in line with NIHR guidance [29].

### Criteria for discontinuing/modifying interventions {11b, 18b, 21b and 22}

Due to the nature of the trial, there are no formal stopping rules as no problems detrimental to the participant are anticipated (supported by data from the hospitalised trial [30]). The intervention will be discontinued if the participant withdraws consent or if a change in circumstance results in the participant becoming unsafe to engage in the intervention. Data collected up until the date of withdrawal will remain in the analysis with participants’ consent.

As the intervention is individualised, any modifications will be determined through discussions between the patient and healthcare professional. Reasonable adaptations to exercise will be made based on clinical judgement and participant need.

Participants will be carefully monitored with particular attention to those displaying signs of post-exertional malaise (PEM) and post-exertional symptom exacerbation (PESE) via the DePaul outcome measure. Steps have been taken to ensure those with known PEM/PESE are not recruited to this trial to ensure their symptoms are not worsened by rehabilitation. If it appears that someone is experiencing PEM/PESE during the intervention phase, then they will be suspended from the trial, upon discussion with the individual, and referred to the appropriate clinic(s).

### Provisions for post-trial care {30}

After involvement in the trial has concluded, individuals allocated to the control group will be offered face-to-face rehabilitation if desired.

### Outcomes {12}

A comprehensive, individualised face-to-face assessment will be conducted for all participants, to include demographics of protected characteristics (to inform the secondary analysis of healthcare inequalities), social history (to include employment status, smoking history), history of presenting complaint (Long Covid history), and comorbidities. Participants’ home postcodes will be indexed to the indices of multiple deprivation (IMD) to inform socioeconomic status. All outcome measures will be performed before and after the 6-week intervention phase. The core outcome set for Long Covid [31] developed through international consensus was consulted to inform which outcome measures were selected for use in this study.

#### Primary outcome

Maximal exercise capacity was measured by absolute change in ISWT distance (in metres) 6 weeks after baseline (±2 weeks). The ISWT will be performed in line with technical standards [32]. In brief, the test is an externally paced, incremental test that requires the patient to walk around a 10-m (m) course at a target speed dictated by audio bleeps. As a maximal, progressive test, the walking speed starts slowly but increases each minute, with the test terminated when the patient is no longer able to keep up with audio cues or by the assessor if the required speed is not attained. The test will be performed twice pre-intervention for familiarisation, with the highest distance achieved used for exercise prescription (ESWT speed). The test will be repeated once after the intervention (Table 1).

**Table 1:** Secondary outcome measures

Acceptability	Physical Measures	Questionnaires
Quantitative measure of completion in the intervention group	Short physical performance battery (SPPB) [33]	Generic HRQoL: EQ-5D-5L [34]
Qualitative evidence via focus groups and semi-structured interviews	Physical activity monitoring: ActiGraph accelerometer device [35]	Fatigue: Functional Assessment of Chronic Illness Therapy Fatigue Scale (FACIT-Fatigue) [36]
	Maximum isometric quadriceps strength (kg) [37]	Depression: Patient Health Questionnaire (PHQ9) [38]
	Endurance Shuttle Walk Test (ESWT) (seconds) [39]	Anxiety: Generalised Anxiety Disorder 7-item scale (GAD7) [40]
		Breathlessness: MRC Dyspnoea scale [41]
		Covid-specific HRQoL: Modified C19-YRS [42]
		Cognitive Function: Montreal Cognitive Assessment (MoCA) [43]
		Post Exertional Malaise: DePaul Symptom Questionnaire Short Form (DSQ-SF) [25]

#### Additional exploratory outcome measures

These will be optional outcome measures for patients, which they can opt into via the consent form. These exploratory measures have been included to build on the insights gained from prior research.

##### Immune Markers {33}

Blood will be collected at baseline and at 6 weeks to assess markers of immune ageing and immune-mediated inflammation including (but not limited to) T-cell surface and intracellular phenotype analysis (with or without stimulation) and circulating biomarkers such as antibodies, cytokines, or other circulating proteins. A total of 50 ml (2 ½ tablespoons) venous blood samples will be taken from the arm by a trained member of the research team at the baseline and 6-week assessment visits. Blood samples will be divided into aliquots. Plasma, serum, and peripheral blood mononuclear cells will be separated. A whole blood sample will also be sent for FBC analysis via routine pathology laboratory analysis.

The samples will be stored at −80 °C (plasma and serum) or −150 °C (PBMCs) confidentially in a coded and anonymised form, in accordance with the Human Tissue Act 2004.

##### Heart Rate Variability

A noninvasive 12-lead ECG Holter device (Norav Medical) will be used. Data will be collected continuously for 24 h from patients in either group before and after the intervention period. In addition, resting 12-lead ECG recordings will be collected for 10 min at baseline and again after the intervention. Biomarkers of interest include QT variability, signal averaging ECG (fragmented QRS complexes), temporal T-wave morphology variability, and T-wave alternans. This will provide a wide analysis of heart rates that are suitable for a detailed assessment of the autonomic function before and after rehabilitation programmes (Table 2).

**Table 2:** Spirit Figure

	STUDY PERIOD		
	Enrolment	Intervention	Post-allocation
TIMEPOINT	0	0–6 weeks	6–8 weeks
**ENROLMENT:**			
Eligibility Screen	X		
Informed Consent	X		
Allocation	X		
**INTERVENTIONS:**			
Rehabilitation		X	
Control		X	
**ASSESSMENTS:**			
Incremental Shuttle Walking Test (ISWT)	X		X
Short Physical Performance Battery (SPPB)	X		X
Quadriceps Strength	X		X
Physical Activity Monitoring	X		X
Endurance Shuttle Walking Test (ESWT)	X		X
EuroQol 5-Dimension 5-Level (EQ-5D-5L)	X		X
Covid-19 Yorkshire Rehabilitation Scale (C19-YRS)	X		X
Patient Health Questionnaire-9 (PHQ-9)	X		X
Generalised Anxiety Disorder-7 (GAD-7)	X		X
Medical Research Council (MRC) Dyspnoea Scale	X		X
Functional Assessment of Chronic Illness Therapy – Fatigue (FACIT-F)	X		X
Montreal Cognitive Assessment (MOCA)	X		X
DePaul Symptom Questionnaire – Short Form (DSQ-SF)	X		X
**OPTIONAL EXPLORATORY MEASURES:**			
Immune Markers	X		X
Heart Rate Variability	X		X

### Uptake, compliance and completion

There will be a screening log recorded of the number of people approached, which will be used to compare with those who uptake and consent.

Quantitative measure of completion in the intervention group will be defined as the number of patients attending 75% of their scheduled sessions (75% selected to align with national rehabilitation guidance [44]) or attended follow-up assessment.

### Sample size {14}

The sample size for the randomised controlled trial is calculated based on the primary outcome of ISWT distance, with a change of 50 m at 90% power, a standard deviation of 17 m, and 0.05 type 1 error. These figures have been previously determined and referenced in the literature as the minimum important difference and variance for the ISWT [10, 45].

The sample size required is based on a power calculation for a two-sample *t*-test. The pwr.t.test function from the pwr package [46], in R, was used to calculate a required sample size of 23 participants per group, 46 participants in total. This has been inflated by 20% to allow for attrition, and, therefore, 56 participants will be recruited.

### Recruitment {15}

Patients will be recruited at the point of referral from the Long Covid Assessment Service into the Long Covid Rehabilitation Service. This referral is only completed when the assessing clinician has deemed them ready and clinically suitable for a rehabilitation programme.

Potential participants may be directly issued a patient information sheet (PIS) by their clinical team and asked if a member of the study team can contact them to discuss the trial either over the phone or via email (as per their preference). Patients invited at the point of referral will use the reply slip on the foot of the PIS and the included prepaid envelope to indicate they wish to be contacted by the study team. Potential participants that self-identify or are referred by their GP will be investigated and, if appropriate, invited to the Long Covid clinic prior to the commencement of the trial.

Personal information will not be accessed or reviewed by the study team until consent to contact has been obtained.

### Randomisation {16a, 16b and 16c}

Due to the sample size, stratification will be used to provide additional assurance that important factors are equally represented in the two trial groups to facilitate comparability [47]. The covariates deemed important in this case to balance are those of sex and age.

Randomisation will be generated through online randomisation software ‘Sealed Envelope’ (https://www.sealedenvelope.com), using block randomisation with stratified blocks of six participants. Researchers involved in delivering the rehabilitation programmes will implement the randomisation on ‘Sealed Envelope’ and assign participants. Individuals who are able to participate will be randomised on a 1:1 ratio to face-to-face rehab (intervention) or control (usual care).

### Blinding {17a and 17b}

The statistician will be blinded, and blinded members of the research team will conduct post-intervention assessments. Due to the nature of the intervention, blinding the patient and wider intervention team will not be possible. The study co-ordinator will conduct baseline assessments and attend the rehabilitation class and therefore will not be blinded. Unblinding may be permissible in the interest of patient safety if there is an adverse event.

### Data management {18a, 19, 27 and 29}

All investigators and study site staff will comply with the requirements of the Data Protection Act 1998 with regard to the collection, storage, processing, and disclosure of personal information. All trial documentation containing identifiable patient data will be managed in accordance with ICH GCP, the UK Policy Framework for Health and Social Care, and the 2018 UK General Data Protection Regulation (GDPR).

The trial personnel will ensure that the participants’ anonymity is maintained. Following consent, each participant will be given a unique participant identifier code to preserve the anonymity of the data.

Quantitative data will be entered into an electronic data capture tool (REDCap) hosted by the BRC. The minimum number of individuals necessary for quality control, audit, and analysis will be granted permission to use this data within the national safe haven. Data will be quality checked by a second researcher, and any outliers will be explored and removed as appropriate. Data validation will take place at the end of the study prior to data lock.

Confidentiality will be maintained for qualitative data, which will be anonymised at the point of transcription, the only reference on the recording being the unique participant identifier. All personal names, place names, and organisation names, and any other data deemed to risk potential identification of a participant, will be anonymised. Audio recordings will be stored securely with password encryption on the electronic site file; once transcriptions are finalised, the files will be deleted.

Only the CI, study coordinator, and statistician will have access to the full dataset. Other members of the research team and wider research community may make a formal data use request in writing to access the full dataset; however, any such requests must be approved by the Trial Management Group. Participating investigators have rights to publish discrete elements of the study data.

### Statistical analysis {20a, 20b and 20c}

The analysis will compare the intervention group to the usual care group. Changes in the primary outcome (ISWT distance) will be compared between the groups using a paired *t*-test (or nonparametric equivalent if the data is not normally distributed). Two sensitivity analyses will be conducted for the primary outcome: (1) Missing data will be imputed using multiple imputation allowing for a full ITT analysis and (2) a per-protocol analysis with those that adhere to the intervention defined if they attended 75% of face-to-face sessions or attended for follow-up appointments.The ISWT will also be analysed using generalised linear mixed models (GLMM); this will allow for in-group adjustments for other variables which may add to the richness of the qualitative data at the point of data mixing.

Secondary outcomes are not powered. All analysis of the primary and secondary outcomes will be performed using R (Version 4.3.1), [48]. Continuous variables will be analysed using the *t*-test or nonparametric equivalent. Categorical variables will be analysed using the chi-squared test or non-parametric equivalent.

Blood biomarkers will be analysed using generalised linear mixed models (GLMM) or similar, with intervention group (control vs face to face), time point (pre vs post), and group × time treated as fixed effects; a random intercept will be included for the participant identifier, and all models will be adjusted for sex and baseline values for each variable.

For the heart rate variability, data will be analysed using robust signal processing approaches by a research associate. Advanced HRV analysis will be performed with the Holter ECGs. Biomarkers of interest include QT variability, signal averaging ECG (fragmented QRS complexes), temporal T-wave morphology variability, and T-wave alternans. These features will be compared and combined to be further correlated with patient-specific characteristics.

GLMMs will be fitted using the lmer function from the lme4 package [49]. Missing data for the primary and secondary outcomes will be imputed using a Bayesian framework. GLMMs will be fitted to outcomes with and without imputation. Imputation was performed using the JointAI library [50].

### Oversight and monitoring {5d, 21a, 22, 23 and 25}

A Trial Management Group comprising of all protocol contributors will meet at least quarterly during the recruitment phase with a view to reviewing targets and progress made. Additional meetings will be arranged as and when required. The group will also discuss interpretation of the findings and dissemination plans for the trial. There will be no data monitoring committee (DMC) due to the low-risk nature of the intervention.

All adverse events arising in either group will be documented and reported. This includes but is not limited to events that require further medical attention or hospitalisation. Adverse events will be recorded in an adverse events or serious adverse events log in the investigator site file and reported to the sponsor. Adverse events will be explored and categorised as related or unrelated to the trial intervention.

Protocol amendments will be submitted to the sponsor for approval prior to submission to the NHS REC. For any amendments, the CI, in agreement with the sponsor, will use the ‘Amendment Tool’ Excel template to categorise the amendment as A, B, or C and describe both the amendment and the rationale. Following completion of the declaration, this will then be submitted online alongside supporting documentation via the IRAS portal for consideration.

After the amendment has been submitted online, it will be shared with relevant personnel at the participating organisation. The email, with the completed and locked Amendment Tool and any amended documents attached, will be forwarded to the University Hospitals of Leicester NHS Trust R&D Office and local research team. Amendments will not be implemented until the approval for the amendment has been issued. Amendment history will be kept within the site file to aid identification of the most recent protocol version. Participants will be re-consented if significant changes are made to the study protocol.

### Patient and public involvement and engagement (PPIE)

Patient and public involvement and engagement (PPIE) is recognised as an essential aspect of the research process. Patients have been involved from conception of trial design and will continue to be involved throughout the delivery phases and to optimise dissemination. This involvement has been shaped by the ‘UK Standards for Public Involvement’ [51]. The research team and PPIE group will continue to work together in a mutually respectful partnership, with contributions recognised and valued and suggestions incorporated. Participants receive high street gift vouchers to the value outlined in the NIHR public contributor payment policy [52], in recognition of their time, acknowledgement in resultant publications, and reimbursement of expenses for attending meetings face to face.

PPIE will continue throughout the recruitment and data collection period by means of a patient advisory group who will meet regularly and a representative who has volunteered to sit as a member of the Trial Management Group (author E. Z.). Advice will be sought on the content of the proposed topic guide, which will be utilised in both qualitative components with respect to accessibility of language, question relevance, and appropriateness.

During the results analysis and dissemination phases, the broader PPIE committee will be consulted and suggestions sought to translate the findings to ensure they are accessible and land appropriately with those who would benefit. This may include plain language communications to support groups and the wider patient community.

## Discussion

The purpose of this study is to determine whether face-to-face exercise-based rehabilitation (‘the intervention’) is effective and acceptable for Long Covid patients who have not been hospitalised, when compared to usual care alone. The mixed-methods research design comprises a randomised controlled trial (RCT) with an embedded qualitative evaluation with framed questions to investigate acceptability. Quantitative data alone would be insufficient to answer the research question; therefore, qualitative and quantitative approaches will be integrated to build a more comprehensive understanding.

There have been attempts to reduce the influence of bias; however, some processes are more difficult to mitigate. For example, it is not possible to mask trial participants or clinical delivery practitioners to treatment allocation, which may bias treatment effect estimates. With regard to selection bias and the recruitment strategy, it is imperative to recruit from the medically led Long Covid assessment service to ensure medical suitability and therefore safety. However, there is research to suggest people with Long Covid feel unsupported, disbelieved, and dismissed by healthcare professionals [53], which may influence who presents to healthcare and are therefore screened for inclusion in this study.

If meeting recruitment targets proves challenging, there are mitigations in place to approach previous patients who have expressed an interest in research and have consented to be contacted about future research opportunities. It is anticipated that rehabilitation drop-outs for the purposes of qualitative interviews will be more challenging to invite; however, there will be an effort made by the researchers to seek further engagement by means of a telephone call for those who consented to be contacted or via their assessing clinician when attending for medical follow-ups.

The substantive contributions of those listed as authors on this protocol meet the criteria outlined by the International Committee of Medical Journal Editors (ICMJE) [54] and will be recognised through the granting of authorship on the final trial report. Professional writers will not be employed to assist at any stage in the research process.

### Dissemination plans {31a, 31b and 31c}

Dissemination will include peer-reviewed publication, conference presentations, presentations to the clinical team, and engagement with the media. In addition, we will disseminate via patient and public involvement and engagement (PPIE) both during and at the end of the project. Author K. K. will draw together an overview of the research process in its entirety and subsequent findings to present to examiners as her thesis. Research participants will receive a lay summary after the results have been published. Anonymised quantitative and qualitative datasets generated will be available upon request (after all publications are completed).

## Trial status

Trial recruitment started in November 2024, and its estimated recruitment will continue until December 2025. The current protocol is version 1.2 of 04-02-2025.

## Supplementary Information

Additional file 1. SPIRIT checklist.

## References

[1] NICE. Long-term effects of coronavirus (long COVID): What is it? 2022 [cited 2024 28th October]; Available from: https://cks.nice.org.uk/topics/long-term-effects-of-coronavirus-long-covid/background-information/definition/.

[2] Augustin M, et al. Post-COVID syndrome in non-hospitalised patients with COVID-19: a longitudinal prospective cohort study. Lancet Reg Health. 2021;6:100122–100122. 10.1016/j.lanepe.2021.100122 PMC8129613 34027514

[3] Natarajan A, et al. A systematic review and meta-analysis of long COVID symptoms. Syst Rev. 2023;12(1):88–88. 37245047 10.1186/s13643-023-02250-0 PMC10220332

[4] Bowyer RCE, et al. Characterising patterns of COVID-19 and long COVID symptoms: evidence from nine UK longitudinal studies. Eur J Epidemiol. 2023;38(2):199–210. 36680646 10.1007/s10654-022-00962-6 PMC9860244

[5] Yang C, Tebbutt SJ. Long COVID: the next public health crisis is already on its way. Lancet Reg Health Europe. 2023;28:100612–100612. 37131860 10.1016/j.lanepe.2023.100612 PMC10006728

[6] Fugazzaro S, et al. Rehabilitation interventions for post-acute COVID-19 syndrome: a systematic review. Int J Environ Res Public Health. 2022. 10.3390/ijerph19095185. 35564579 10.3390/ijerph19095185 PMC9104923

[7] Halabchi F, et al. The effect of exercise rehabilitation on COVID-19 outcomes: a systematic review of observational and intervention studies. Sport Sci Health. 2022;18(4):1201–19. 35789736 10.1007/s11332-022-00966-5 PMC9244056

[8] Lloyd-Evans PHI, et al. Early experiences of the Your COVID Recovery® digital programme for individuals with long COVID. BMJ Open Respir Res. 2022;9(1):e001237. 36171050 10.1136/bmjresp-2022-001237 PMC9527747

[9] Daynes E, et al. Early experiences of rehabilitation for individuals post-COVID to improve fatigue, breathlessness exercise capacity and cognition – a cohort study. Chronic Resp Dis. 2021;18:14799731211015692–14799731211015692. 10.1177/14799731211015691 PMC8114752 33957805

[10] Daynes E, et al. Post-hospitalisation COVID-19 rehabilitation (PHOSP-R): a randomised controlled trial of exercise-based rehabilitation. Eur Respir J. 2025. 10.1183/13993003.02152-2024. 39978856 10.1183/13993003.02152-2024 PMC12095904

[11] McGregor G, et al. Clinical effectiveness of an online supervised group physical and mental health rehabilitation programme for adults with post-covid-19 condition (REGAIN study): multicentre randomised controlled trial. BMJ. 2024;384:e076506–e076506. 38325873 10.1136/bmj-2023-076506 PMC11134408

[12] NICE RCGP, SIGN. COVID-19 rapid guideline: managing the longterm effects of COVID-19 Version 1.20 2022 [cited 2024 2nd January]; Available from: https://www.nice.org.uk/guidance/ng188/resources/covid19-rapid-guideline-managing-the-longterm-effects-of-covid19-pdf-51035515742.

[13] NHS England. The NHS plan for improving Long COVID services. Publication approval reference: C1607 2022 [cited 2024 2nd January]; Available from: https://www.england.nhs.uk/wp-content/uploads/2022/07/C1607_The-NHS-plan-for-improving-long-COVID-services_July-2022.pdf.

[14] NHS England. Commissioning guidance for Post COVID services for adults, children, and young people. 2023 [cited 2024 2nd January ]; Available from: https://www.england.nhs.uk/long-read/commissioning-guidance-for-post-covid-services-for-adults-children-and-young-people/.

[15] Houchen-Wolloff L, et al. A typology of healthcare pathways after hospital discharge for adults with COVID-19: the evolution of UK services during pandemic conditions. ERJ Open Res. 2023;9(4):565–2022. 10.1183/23120541.00565-2022 PMC10423987 37583962

[16] Wolf S, Zechmeister-Koss I, Erdös J. Possible long COVID healthcare pathways: a scoping review. BMC Health Serv Res. 2022;22(1):1–1076. 35999605 10.1186/s12913-022-08384-6 PMC9396575

[17] Buttery S, et al. Patient symptoms and experience following COVID-19: results from a UK-wide survey. BMJ Open Respir Res. 2021;8(1):e001075. 34732518 10.1136/bmjresp-2021-001075 PMC8572361

[18] Brehon K, et al. None of us are lying": an interpretive description of the search for legitimacy and the journey to access quality health services by individuals living with Long COVID. BMC Health Serv Res. 2023;23(1):1396. 38087299 10.1186/s12913-023-10288-y PMC10714615

[19] Alwan NA, et al. Long Covid active case finding study protocol: a co-produced community-based pilot within the STIMULATE-ICP study (Symptoms, Trajectory, Inequalities and Management: Understanding Long-COVID to Address and Transform Existing Integrated Care Pathways). PLoS ONE. 2023;18(7):e0284297–e0284297. 37471432 10.1371/journal.pone.0284297 PMC10358953

[20] Khunti K, et al. Is ethnicity linked to incidence or outcomes of covid-19? BMJ (Online). 2020;369:m1548–m1548. 32312785 10.1136/bmj.m1548

[21] Shabnam S, et al. Socioeconomic inequalities of long COVID: a retrospective population-based cohort study in the United Kingdom. J R Soc Med. 2023;116(8):263–73. 37164035 10.1177/01410768231168377 PMC10469969

[22] Singh SJ, et al. Balancing the value and risk of exercise-based therapy post-COVID-19: a narrative review. Eur Respir Rev. 2023. 10.1183/16000617.0110-2023. 38123233 10.1183/16000617.0110-2023 PMC10731468

[23] American College of Sports M. Acsm's Guidelines for Exercise Testing and Prescription. 2022.

[24] National Institute for Health and Care Excellence. COVID-19 rapid guideline: managing the long-term effects of COVID 19. [NG191] 2021 [cited 2024 26th January]; Available from: https://www.nice.org.uk/guidance/ng191. 33555768

[25] Sunnquist M, et al. The development of a short form of the DePaul symptom questionnaire. Rehabil Psychol. 2019;64(4):453–62. 31318234 10.1037/rep0000285 PMC6803042

[26] Mahler DA, Horowitz MB. Perception of breathlessness during exercise in patients with respiratory disease. Med Sci Sports Exerc. 1994;26(9):1078–81. 7808239

[27] Borg GAV. Psychophysical bases of perceived exertion. Med Sci Sports Exerc. 1982;14(5):377–81. 7154893

[28] Sekhon M, Cartwright M, Francis JJ. Acceptability of healthcare interventions: an overview of reviews and development of a theoretical framework. BMC Health Serv Res. 2017;17(1):88–88. 28126032 10.1186/s12913-017-2031-8 PMC5267473

[29] NIHR. Payment guidance for researchers and professionals. 2022 [cited 2024 4th March]; Version 1.4 - July 2023: Available from: https://www.nihr.ac.uk/documents/payment-guidance-for-researchers-and-professionals/27392.

[30] Daynes E, et al. The effect of COVID rehabilitation for ongoing symptoms post hospitAlisation with COVID-19 (PHOSP-R): protocol for a randomised parallel group controlled trial on behalf of the PHOSP consortium. Trials. 2023;24(1):61–61. 36703183 10.1186/s13063-023-07093-7 PMC9879232

[31] Gorst SL, et al. Core outcome measurement instruments for use in clinical and research settings for adults with post-COVID-19 condition: an international Delphi consensus study. Lancet Respir Med. 2023;11(12):1101–14. 37926103 10.1016/S2213-2600(23)00370-3

[32] Singh SJ, et al. Development of a shuttle walking test of disability in patients with chronic airways obstruction. Thorax. 1992;47(12):1019–24. 1494764 10.1136/thx.47.12.1019 PMC1021093

[33] Guralnik JM, et al. A short physical performance battery assessing lower extremity function: association with self-reported disability and prediction of mortality and nursing home admission. J Gerontol. 1994;49(2):M85–94. 8126356 10.1093/geronj/49.2.m85

[34] Herdman M, et al. Development and preliminary testing of the new five-level version of EQ-5D (EQ-5D-5L). Qual Life Res. 2011;20(10):1727–36. 21479777 10.1007/s11136-011-9903-x PMC3220807

[35] Aadland E, Ylvisåker E, López Lluch G. Reliability of the actigraph GT3X+ accelerometer in adults under free-living conditions. PLoS ONE. 2015;10(8):e0134606–e0134606. 26274586 10.1371/journal.pone.0134606 PMC4537282

[36] Smith EMD, Lai J-SP, Cella DP. Building a measure of fatigue: the functional assessment of chronic illness therapy fatigue scale. PM & R. 2010;2(5):359–63. 20656617 10.1016/j.pmrj.2010.04.017

[37] Edwards RHT, et al. Human skeletal muscle function: description of tests and normal values. Clin Sci Mol Med. 1977;52:283–90. 844260 10.1042/cs0520283

[38] Kroenke K, Spitzer RL, Williams JBW. The PHQ-9: validity of a brief depression severity measure. J Gen Int Med: JGIM. 2001;16(9):606–13. 10.1046/j.1525-1497.2001.016009606.x PMC1495268 11556941

[39] Revill SM, et al. The endurance shuttle walk : a new field test for the assessment of endurance capacity in chronic obstructive pulmonary disease. Thorax. 1999;54(3):213–22. 10325896 10.1136/thx.54.3.213 PMC1745445

[40] Spitzer RL, et al. A Brief Measure for Assessing Generalized Anxiety Disorder: The GAD-7. Arch Int Med (1960). 2006;166(10):1092-1097. 10.1001/archinte.166.10.1092 16717171

[41] Fletcher CM, et al. Significance of respiratory symptoms and the diagnosis of chronic bronchitis in a working population. BMJ. 1959;2(5147):257–66. 13823475 10.1136/bmj.2.5147.257 PMC1990153

[42] Sivan M, et al. The modified COVID-19 Yorkshire rehabilitation scale (C19-YRSm) patient-reported outcome measure for long Covid or post-COVID-19 syndrome. J Med Virol. 2022;94(9):4253–64. 35603810 10.1002/jmv.27878 PMC9348420

[43] Nasreddine ZS, et al. The Montreal Cognitive Assessment, MoCA: a brief screening tool for mild cognitive impairment. J Am Geriatr Soc. 2005;53(4):695. 15817019 10.1111/j.1532-5415.2005.53221.x

[44] NHS Medical Directorate. Service Specification: Pulmonary Rehabilitation Service. 2012 [cited 2025 17th April]; Available from: https://assets.publishing.service.gov.uk/media/5a74951e40f0b61938c7e9ed/Service-Spec-Pulmonary-rehabilitation.doc.

[45] Singh SJ, et al. Minimum clinically important improvement for the incremental shuttle walking test. Thorax. 2008;63(9):775–7. 18390634 10.1136/thx.2007.081208

[46] Champely S. pwr: Basic Functions for Power Analysis. 2020 [cited 2024 8th March]; Available from: https://cran.r-project.org/web/packages/pwr/.

[47] FDA. Good Review Practice: Clinical Review of Investigational New Drug Applications. 2013 [cited 2024 15th February]; Available from: https://www.fda.gov/media/87621/download.

[48] R Core Team. R: A Language and Environment for Statistical Computing 2023; Available from: https://www.R-project.org.

[49] Bates D, et al. Fitting linear mixed-effects models using lme4. J Stat Softw. 2015;67(1):1–48.

[50] Erler NS, Rizopoulos D, Lesaffre EMEH. JointAI : Joint analysis and imputation of incomplete data in R. J Stat Softw. 2021;100(20):1–56.

[51] NIHR. UK Standards for Public Involvement. 2019 [cited 2024 19th January]; Available from: https://sites.google.com/nihr.ac.uk/pi-standards/standards/inclusive-opportunities.

[52] NIHR. NIHR public contributor payment policy. 2022 [cited 2024 16th April]; Version 4.0 - September 2022:[Available from: https://www.nihr.ac.uk/documents/nihr-public-contributor-payment-policy/31626.

[53] Pearson M, et al. Creative long covid: a qualitative exploration of the experience of long covid through the medium of creative narratives. Health Expect. 2022;25(6):2950–9. 36148648 10.1111/hex.13602 PMC9700147

[54] International Committee of Medical Journal Editors (ICMJE). Defining the Role of Authors and Contributors. 2024 [cited 2024 13th February]; Available from: https://icmje.org/recommendations/browse/roles-and-responsibilities/defining-the-role-of-authors-and-contributors.html#:~:text=2.-,Who%20Is%20an%20Author%3F,for%20important%20intellectual%20content%3B%20AND.

